# A Latent Class Analysis of Student Eye Care Behavior: Evidence From a Sample of 6–17 Years Old in China

**DOI:** 10.3389/fpubh.2022.914592

**Published:** 2022-06-15

**Authors:** Mengying Li, Wenjing Wang, Boya Zhu, Xiaodong Tan

**Affiliations:** School of Public Health, Wuhan University, Wuhan, China

**Keywords:** adolescent, latent class analysis, visual health management, myopia, eye care

## Abstract

**Purpose:**

To understand the latent classes and distribution of an adolescent eye care behavior, and to provide a basis for the formulation of appropriate adolescent vision health management interventions.

**Methods:**

Information on eye behavior and eye health of primary and secondary school students in Wuhan was collected by multistage stratified cluster sampling. The latent class analysis (LCA) method was used to analyze the students' eye care behavior, and the latent class model (LCM) was built.

**Results:**

A total of 6,130 students were enrolled in this study, of which 53.56% were males, aged from 6 to 17 years old, with an average age of 10.33 ± 2.60. The latent class results classified the adolescents' eye care behaviors into bad behaviors, moderate behaviors, and healthy behaviors. The model fitting results were as follows: Akaike Information Criterion (AIC) was 36,698.216, Bayesian Information Criterion (BIC) was 36,906.565, Adjusted Bayesian Information Criterion (aBIC) was 36,808.056, and entropy was 0.838.Compared with the healthy behaviors class, the bad behaviors class was more prevalent in high schools (*p* = 0.003), non-demonstration schools (*p* = 0.001), and most of this group had astigmatism (*p* = 0.002). The moderate behaviors class predominately consisted of females (*p* = 0.001), 15–17 years old (*p* = 0.005, 6~8 years old as the reference), from non-demonstration schools (*p* < 0.001), and most had myopia (*p* = 0.009).

**Conclusion:**

There were differences in basic demographic characteristics, visual acuity development level, and family visual environment among different classes. In the management and intervention of an adolescent vision health, we should continue to promote the visual health management of adolescents based on visual monitoring and realize the early intervention and guidance of individuals in bad behaviors class.

## Introduction

Visual health refers to normal visual physiology and visual psychology and good visual social adaptation on the premise of not suffering from eye diseases and abnormal symptoms such as visual fatigue. An analysis of studies suggests that by 2050, nearly half of the world's population may be myopic, with up to 10% being highly myopic (1).The World Health Organization (WHO) lists myopia as one of the 5 eye diseases requiring priority elimination and improvement (2). At the same time, there are racial and ethnic differences in the levels and prevalence of myopia, both of which are higher in Asia than in other parts of the world (3).At present, the prevalence of myopia among adolescents in China is characterized by a high prevalence (4, 5), fast growth rate (6, 7), and early age onset (8). China has the most teenage myopia patients in the world (9). Some researchers predict that if no intervention measures are taken, the myopia rate of Chinese adolescents will reach 61.8% in 2030 (10).

Comprehensive eye care (CEC) aims to ensure that people have access to the ophthalmic health services that meet the needs of each stage of their lives, which includes visual loss prevention, due to poor eye care habits and behavior (11, 12). Although a small percentage of myopia is inherited, much more is simply caused by poor eye care habits and behavior (13–16). A large number of studies have shown that near work, incorrect reading, and writing posture, and prolonged use of electronic devices can lead to visual fatigue, altered refractive state, and myopia (17–20). In terms of daily life, sleep deprivation is a risk factor for the development of myopia in teenagers (21). A diet high in sugar and cholesterol can also contribute to myopia (22, 23). There is a wealth of epidemiological evidence about the amount of time spent outdoors, indicating that adequate time spent outdoors is one of the most important factors in protecting visual health (24–26), which may be due to vitamin D and dopamine (27–29). Given the close relationship between behavior and visual health (30), it is necessary to effectively identify the accumulation of vision-related risks in adolescents by exploring and studying heterogeneous subsets of related behaviors.

Although most studies have shown that visual health development is significantly correlated with behavior, most of these studies have grouped adolescents according to gender, age, and other conditions for analysis, and it is impossible to judge whether subgroups can be defined only by significant variables. To explore the visual health and behavioral development of adolescents, Wuhan city has carried out visual health management and monitoring for primary and secondary school students. In this study, a latent class model was established to determine the class attributes of adolescents' eye health behaviors and analyze their distribution characteristics, providing a scientific basis for understanding the relationship between adolescents' eye care changes and visual health development.

## Methods

### Study Population

Data were collected from the vision prevention and treatment project for adolescents in Wuhan, which was reviewed and approved by the Ethics Committee of the School of Public Health, Wuhan University. This study was conducted in 2019 and used a multistage stratified cluster sampling approach to recruit participants. According to the basic information released by the Wuhan Education Bureau in 2017, there are 735,799 students in Wuhan. The sample size of the sample survey is calculated as follows:


(1) $$\begin{array}{l} n\geq \frac{N}{{\left(\frac{\alpha }{k}\right)}^{2}\frac{N-1}{P\left(P-1\right)}+1} \end{array}$$

*N* is the total sample number, and *P* is denoted as 0.50. If the sample population is large, the sampling size formula can be written as:


(2) $$\begin{array}{l} n\geq {\left(\frac{k}{\alpha }\right)}^{2}P\left(1-P\right) \end{array}$$

In general, α is denoted as 0.05 and *K* as 1.96. According to the statistical formula, it was estimated that 385 participants in each group were required. Considering grade differences, the sample size of this study is *n*≥12(grade)^*^385 ≈ 4,620, which means that the sample size needs to be >4,620 people.

Schools are divided by the Wuhan Education Bureau into vision health management demonstration schools and non-demonstration schools, and the classification standards are as follows: (1) Whether to carry out regular visual health management; (2) Whether to successfully apply for a municipal demonstration school; if both standards are met, the school will be regarded as a demonstration school of visual health. In consideration of geographical location (urban/rural region), whether it is a demonstration school or not, and the key age of myopia prevention and control, 140 schools in 14 districts (such as primary school, junior high school, and senior high school) were selected for this study, and a total of 6,130 students were enrolled.

This study adopted a self-made questionnaire as a survey tool, which development took reference from the Questionnaire of Vision Care Related Behavior for Students (AQVCRBS) (31). The results were filled in by students through the “Internet +” vision monitoring management application platform. The survey content included general demographic characteristics (sex, age, residence, education stage, school type);and eye care behaviors (near work, reading posture, time of electronics use, duration of sleep, eating habits, supplementation of vitamin A, outdoor exercise, eye exercises, non-sports training courses, eye muscle exercises). Each respondent completed a self-report questionnaire independently, and both the respondent and guardian signed informed consent forms.

### Inclusion and Exclusion Criteria

The inclusion criteria for participants were as follows: (1) Students aged 6–17 years. (2) The legal guardian signed the informed consent. (3) Students without congenital eye diseases, such as congenital brain damage and visual impairment. (4)No neurological disorders, such as severe cognitive impairment.

The exclusion criteria for participants were as follows: (1) The legal guardian did not consent to participate in the vision test or related investigation. (2) Students with an incomplete investigation.

### Examination Method

The results of vision monitoring will be reported by each school through the “Internet +” vision monitoring management platform and sent to the Wuhan Visual Prevention and Control Center. Visual acuity assessment uses the flat vision examination instrument, which has passed the approval and detection of relevant departments. Refractive inspection was performed according to the recommended desktop automatic computer optometry, and optometry equipment by the standard (ISO10342ophthalmic instrument-optometry) provisions. All physicians or investigators will be trained to independently perform standard ophthalmic examinations.

### Diagnostic Criteria

In this study, the spherical equivalent (SE) was calculated as the dioptric powers of the sphere and half of the cylinder (sphere+0.5 cylinders). Myopia was defined as SE of <0.5 diopter (D) and visual acuity <5.0. Astigmatism is the diopter difference between 2 main diameters of the same eye (absolute diopter value of the column mirror) above 0.50D.

### Data Analysis

In this study, each item of students' eye care behaviors in 2019 was parameterized by latent class analysis (LCA), and the latent class model (LCM) was constructed, which is a statistical analysis that addresses the relationship between types of latent variables. The optimal model is determined by the following criteria: Akaike information criterion (AIC), Bayesian information criterion (BIC), sample-size adjusted Bayesian information criterion (aBIC), Bootstrap likelihood ratio test (*BLRT*), and adjusted Lo-Mendell-Rubin likelihood ratio test (*aLMR*). After identifying the latent classes, the regression mixture modeling (RMM) was used to analyze the sociodemographic characteristics and visual health levels of different behavioral groups. SPSS 25.0 and Mplus 7.4 statistical software were used to analyze the data, and *p* < 0.05 was taken as the criterion of significance.

## Results

### Demographic Characteristics

**Figure 1:**
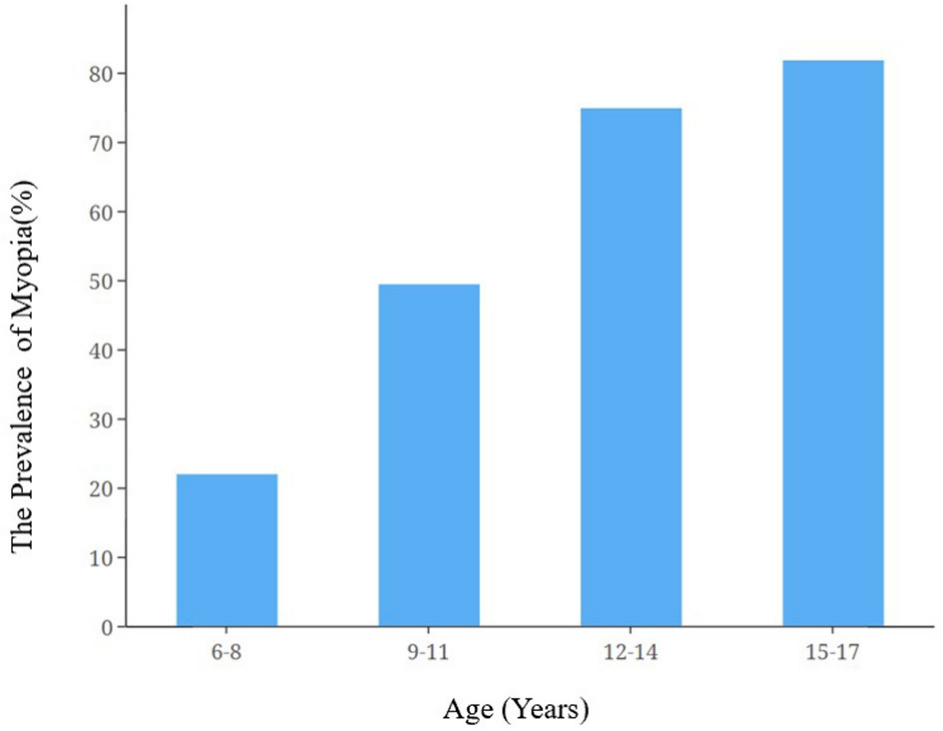
Bar chart of the prevalence of myopia among adolescents of different ages.

A total of 7,840 primary and secondary school students were recruited for this study, among which 1.710 were excluded (21.81%) due to transfer to other schools, incomplete questionnaires, and other reasons. Table 1 contains basic demographic characteristics. Among the participants, 6,130 were included in the final analysis, of which 3,283 (53.56%) were men and 2,847 (46.44%) were women. The sample population was 6–17 years old, with an average age of 10.33±2.60.Respondents aged 6–8 years accounted for the highest proportion (37.63%), and those aged 15–17 years accounted for the lowest proportion (10.73%). In terms of education, primary school students accounted for the highest proportion (56.51%), and the proportion of high school students was the lowest at 13.16%. The number of myopic students in the sample population was 3,067, accounting for 50.03%. The prevalence of myopia increased as age increased (Figure 1). There were statistically significant differences in the prevalence rate of myopia among students with different behaviors (Table 2).

**Table 1:** Distribution of basic demographic characteristics of participants.

**Variables**	**Number**	**%**
Sex
Male	3,283	53.56
Female	2,847	46.44
Age(year)		
6–8	2,307	37.63
9–11	1,360	22.19
12–14	1,805	29.45
15–17	658	10.73
Education stage
Primary school	3,464	56.51
Junior high school	1,859	30.33
High school	807	13.16
Type of school
Demonstration school	3,667	59.82
Non-demonstration school	2,463	40.18
Urban/rural region
Central urban area	3,396	55.40
Rural-urban area	2,734	44.60
Myopia		
Yes	3,067	50.03
No	3,063	49.96
Astigmatism		
Yes	3,173	51.76
No	2,957	48.24
Wear glasses		
Yes	1,465	23.90
No	4,665	76.10

**Table 2:** Distribution of myopia rate in different eye hygiene behaviors (*N* = 6,130).

**Variables**	**Number**	**%**	**χ^2^**	***p* value**
Q1 Constant close eye contact for more than 40 min				
Yes	2,030	53.86	57.35	<0.001
No	1,037	43.92		
Q2 Hold a pen, read and write correctly				
Yes	1,255	40.34	237.34	<0.001
No	1,812	60.02		
Q3 Regularly use electronic devices for more than 30 min				
Yes	1,715	65.56	439.99	<0.001
No	1,352	38.47		
Q4 Get enough sleep each day				
Yes	1,398	35.21	991.51	<0.001
No	1,669	77.30		
Q5 Poor eating habits				
Yes	1,834	65.92	514.52	<0.001
No	1,233	36.83		
Q6 Pay attention to supplement foods rich in vitamin A				
Yes	1,342	44.25	80.39	<0.001
No	1,725	55.70		
Q7 Outdoor exercise time up to 2 h a day				
Yes	1,158	38.13	341.11	<0.001
No	1,909	61.72		
Q8 Do eye exercises every day				
Yes	1,379	41.19	230.80	<0.001
No	1,688	60.68		
Q9 Attend non-sports training courses regularly				
Yes	1,657	55.29	64.80	<0.001
No	1,410	45.00		
Q10 Exercise or train eye muscles regularly				
Yes	912	42.24	80.92	<0.001
No	2,155	52.26		

### Latent Class Analysis of Eye Care Behavior

Table 3 shows that the information criteria indices AIC, BIC, and aBIC decreased with the increase in the number of latent classes, and reached the maximum value in model 5. From the likelihood ratio test statistics, the entropy value reached 0.838 in model 3, indicating that the model was the most accurate for sample classification when there were 3 latent classes. Based on the model fitting evaluation results and conditional probability distribution of the latent class, the latent class of adolescent visual behavior was finally divided into 3 classes: class 1, class 2, and class 3.

**Table 3:** Results of Latent Class Model (LCM) fitting information.

**Model**	**AIC**	**BIC**	**aBIC**	***Entropy***	***BLRT p* value**	***aLMR*** ***p* value**
1	37,630.335	37,677.381	37,655.137	1.000	-	-
2	36,827.359	36,981.941	36,908.854	0.761	<0.001	<0.001
3	36,698.216	36,906.565	36,808.056	0.838	<0.001	<0.001
4	36,609.493	36,871.610	36,747.679	0.764	0.001	0.014
5	36,525.758	36,841.642	36,692.289	0.735	0.002	0.036

*AIC, Akaike Information Criterion; BIC, Bayesian Information Criterion; aBIC, Adjusted Bayesian Information Criterion; BLRT, Bootstrap Likelihood Ratio Test; aLMR, Adjusted Lo-Mendell-Rubin Likelihood Ratio Test*.

**Figure 2:**
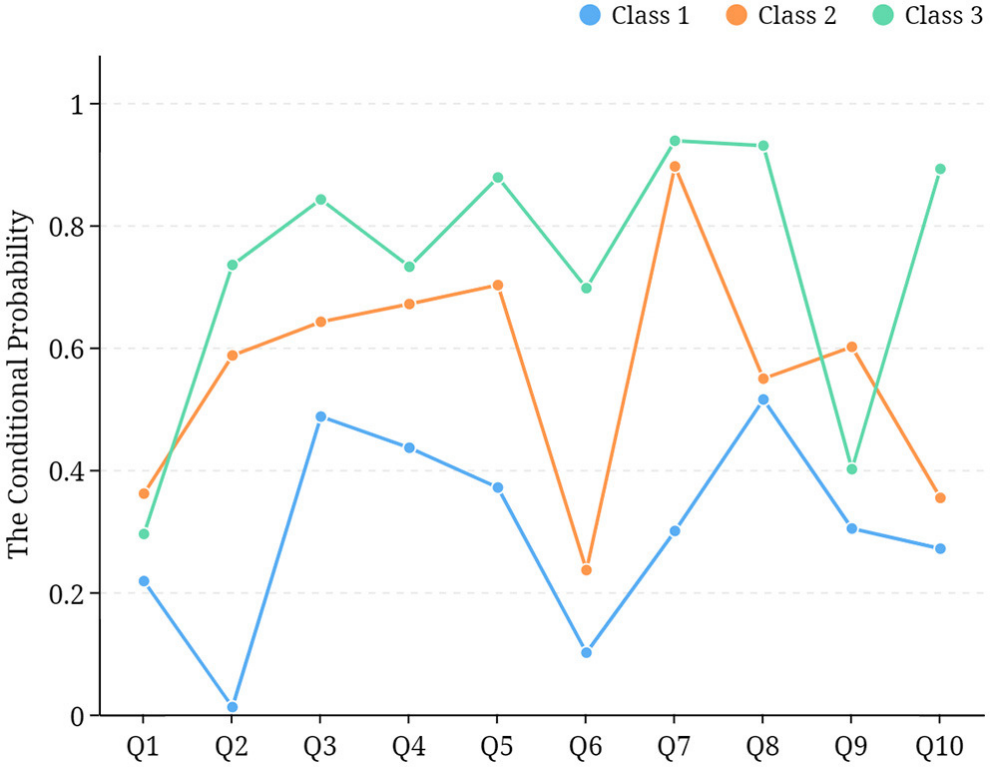
Line chart of latent classes of adolescent eye care behavior. *Q1: Frequent close eye contact for more than 40 min; Q2: Hold a pen, read, and write correctly; Q3: Regularly use electronic devices for more than 30 min; Q4: Get enough sleep each day; Q5: Poor eating habits; Q6: Pay attention to supplement foods rich in vitamin A; Q7: Outdoor exercise time up to 2 h a day; Q8: Do eye exercises every day; Q9: Attend non-sports training courses regularly; Q10: Exercise or train eye muscles regularly. Q1, Q3, Q5 and Q9 were reverse scored.

Table 4 and Figure 2 show the conditional probability of the latent class of adolescent eye care behavior. In class 1, the item probability of frequent use of the eyes for more than 40 min (78.2%) was the highest, and the item probability of holding a pen, reading, and writing correctly (1.2%) was the lowest. In class 3, the item probability of regularly using electronic devices for more than 30 min (15.8%) was the lowest, but outdoor exercise time reached up to 2 h a day (93.8%), and eye exercises were performed every day (93.0%). Compared with the other 2 classes, the conditional probability of class 2 tends to be in the middle. Therefore, class 1 was named the “bad behaviors class,” class 2 was the “moderate behaviors class,” and class 3 was the “healthy behaviors class.”

**Table 4:** The conditional probability of the latent class of adolescent eye care behavior.

**Variables**	**Class 1**	**Class 2**	**Class 3**
Q1. Frequent close eye contact for more than 40 min	0.782	0.639	0.705
Q2. Hold a pen, read and write correctly	0.012	0.587	0.735
Q3. Regularly use electronics for more than 30 min	0.513	0.358	0.158
Q4. Get enough sleep each day	0.436	0.671	0.732
Q5. Poor eating habits	0.629	0.298	0.122
Q6. Pay attention to supplement foods rich in vitamin A	0.101	0.236	0.697
Q7. Outdoor exercise time up to 2 h a day	0.300	0.896	0.938
Q8. Do eye exercises every day	0.515	0.549	0.930
Q9. Attend non-sports training courses regularly	0.696	0.399	0.599
Q10. Exercise or train eye muscles regularly	0.271	0.354	0.892

### Results of Univariate Analysis

Table 5 shows the influence of basic demographic characteristics and visual development on the distribution of latent classes of adolescent eye care behaviors.

**Table 5:** Influence of latent class distribution on adolescent eye care behavior.

**Variables**	**Bad behaviors class (%)**	**Moderate behaviors class (%)**	**Health behaviors class(%)**	**χ^2^**	***p*-value**
Gender				
Male	603 (18.37)	257 (7.83)	2,423 (73.80)	8.25	0.016
Female	476 (16.72)	275 (9.66)	2,096 (73.62)		
Age (year)				
6–8	388 (16.82)	203 (8.80)	1,716 (74.38)	67.43	<0.001
9–11	229 (16.84)	92 (6.76)	1,039 (76.40)		
12–14	286 (16.01)	153 (8.48)	1,363 (75.51)		
15–17	173 (26.29)	84 (12.77)	401 (60.94)		
Education stage				
Primary school	561 (16.20)	289 (8.34)	2,614 (75.46)	54.40	<0.001
Junior high school	313 (16.84)	151 (8.12)	1,395 (75.04)		
High school	205 (25.40)	92 (11.40)	510 (63.20)		
Type of school				
Demonstration school	608 (16.58)	225 (6.14)	2,834 (77.28)	89.14	<0.001
Non-model school	471 (19.12)	307 (12.46)	1,685 (68.41)		
Urban/rural region				
Central urban area	559 (16.46)	294 (8.66)	2,543 (74.88)	7.04	0.030
Rural-urban area	520 (19.02)	238 (8.71)	1,976 (72.28)		
Myopia				
Yes	534 (17.41)	300 (9.78)	2,233 (72.81)	9.42	0.009
No	545 (17.79)	232 (7.57)	2,286 (74.63)		
Astigmatism				
Yes	582 (18.34)	261 (8.23)	2,330 (73.43)	3.68	0.159
No	497 (16.81)	271 (9.16)	2,189 (74.03)		
Wearing glasses				
Yes	379 (25.87)	137 (9.35)	949 (64.78)	96.65	<0.001
No	700 (15.01)	395 (8.47)	3,570 (76.25)		

Of the sociodemographic characteristics, the distribution of gender (*p* = 0.016), age (*p* < 0.001), education stage (*p* < 0.001), type of school (*p* < 0.001), and urban/rural region (*p* = 0.030) differed among the classes of students. In addition, the distributions of myopia (*p* = 0.009) and wearing glasses (*p* < 0.001) were also significantly different among the 3 classes of students.

### Results of the Regression Mixed Model

The results of multicollinearity diagnosis showed that the tolerance between the respective variables was >0.1, and the variance inflation factor (VIF) was <10 (Table 6).The results of the parallel line test showed that *X*^2^ = 210.070, *p* < 0.05. These results showed that polynomial logistic regression can be used.

**Table 6:** Multicollinearity diagnosis of influencing factors of adolescent eye care behavior.

**Variables**	**Collinearity statistics**	
	**Tolerance**	**VIF**
Gender	0.10	1.01
Age	0.17	5.81
Education stage	0.15	6.55
Type of school	0.83	1.21
Urban/rural region	0.98	1.02
Myopia	0.74	1.36
Astigmatism	0.97	1.03
Wear glasses	0.72	1.39

In the bad behaviors class (reference: health behavior class), in terms of visual acuity development status, those with astigmatism was 1.26 (*p* = 0.002) times more likely to be found in the bad behaviors class than in the healthy behaviors class, and those with glasses were 1.90 times more likely to be found in the bad behaviors class than the healthy behaviors class (*p* < 0.001). In addition, the probability of the high school group being distributed in the bad behaviors class was 2.44 (1/0.41, *p* = 0.003) times that of the primary school group, and the probability of a non-demonstration school population in the bad behaviors class was 1.27 times higher than that in the healthy behaviors class (1/0.79, *p* = 0.001).

In the moderate behaviors class (reference: health behavior class), those with myopia were 1.83 times more likely to be found in the moderate behaviors class than in the non-myopic group (*p* = 0.009).In terms of gender, females were 1.35 times more likely to be in the moderate behaviors class than males (1/0.74, *p* = 0.001).Compared with those aged 6–8 years old, those aged 15–17 years old were 5.26 (1/0.19, *p* = 0.005) times more likely to be found in the moderate behaviors class than the healthy behaviors class. Regarding the type of school, people from non-demonstration schools were 2.08 (1/0.48, *p* < 0.001) times more likely to be found in the moderate behaviors class than those from demonstration schools. These results are shown in Table 7.

**Table 7:** Results of a regressive mixed analysis of adolescent eye care behavior.

**Variables**	**Bad behaviors classes** **(Refer to the health behaviors class)**		**Moderate behaviors classes** **(Refer to the health behaviors class)**	
	**OR**	***p value***	**OR**	***p value***
Gender (reference:Female)				
Male	1.11	0.153	0.74	0.001
	(0.96, 1.28)		(0.61, 0.89)	
Age (reference: 15–17 years)				
6–8 years	1.80	0.059	0.19	0.005
	(0.98, 3.30)		(0.06, 0.61)	
9–11years	1.44	0.209	0.17	0.002
	(0.81, 2.56)		(0.05, 0.53)	
12–14years	0.64	0.050	0.35	0.007
	(0.41, 0.10)		(0.16, 0.75)	
Education stage (reference: Senior high school)				
Primary school	0.41	0.003	2.52	0.124
	(0.23, 0.74)		(0.78, 3.19)	
Junior high school	0.89	0.608	1.51	0.294
	(0.58, 1.38)		(0.70, 3.23)	
Type of school (reference: Non- demonstration School)				
Demonstration School	0.79	0.001	0.48	<0.001
	(0.68, 0.91)		(0.38, 0.58)	
Urban/rural region (reference: Rural-urban area)
Central urban area	1.04	0.657	1.16	0.166
	(0.89, 1.21)		(0.94, 1.43)	
Myopia (reference: No)
Yes	0.89	0.185	1.83	0.009
	(0.76, 1.06)		(1.33, 2.54)	
Astigmatism (reference: No)
Yes	1.26	0.002	0.87	0.156
	(1.09, 1.46)		(0.71, 1.06)	
Wear glasses (reference: No)
Yes	1.90	<0.001	1.22	0.157
	(1.57, 2.27)		(0.93, 1.60)	

## Discussion

First, there were 3 subgroups among adolescent eye care behaviors, such as the bad behaviors class, moderate behaviors class, and health behaviors class. Second, the results of regression mixed analysis showed that those from the lower grade group, the demonstration school, and those with good vision were more likely to be distributed in the healthy behavior group.

The latent classes of adolescent behavior vary according to the findings of different researchers. As a survey on adolescent health risks in China in 2020, the Health Risk Behavior Assessment Questionnaire (HRBAQ) was used to analyze the latent class of 22,628 middle school students in China, and found 4 latent classes, such as low-risk classes (64.0%), medium risk class 1 (4.5%), medium risk class 2 (28.8%), and high-risk class (2.7%) (32).Our results were similar. According to the results of the Australian student health behavior survey in 2019, 1,965 students in Australia were divided into 3 latent classes, namely unhealthy class (11.2%), moderate class (40.2%), and healthy class (48.6%), based on their diet, exercise, and sleep habits. This study explored the latent categories of adolescents from the perspective of eye care behavior, which enriches the existing literature on adolescent behavior (33).

In the 3 latent classes of adolescents in Wuhan, class 1 had the highest conditional probability in the items of “Frequent near work” and “Regularly use electronic devices.” Multiple studies have reported that near work and prolonged use of electronic devices significantly increase the risk of myopia (34–36). Therefore, class 1 was named the bad behaviors class because of its weak visual health management ability and high probability of bad behavior. Class 3 was named the healthy behaviors class because it had a higher conditional probability of the positive items. However, the healthy behaviors class was slightly higher than the moderate behaviors class in terms of the measurement items of “Regularly participating in non-sports excellent training courses,” which may indicate that on the one hand, adolescents in the healthy behaviors class attach more importance to the cultivation of healthy behavior and can consciously manage vision health. On the other hand, it also indicates that adolescents are still under great pressure from extracurricular tutoring. Although the adolescents in this class consciously carried out self-vision management, they still had some negative eye care behaviors due to academic pressure.

There were significant differences in basic demographic characteristics among the different classes. Compared with the healthy behaviors class, the bad behaviors class was more distributed in high school and non-demonstration schools, while the moderate behavior group was more distributed in female, 15–17 years old, and non-demonstration schools. These findings suggested that with the increase in age and academic pressure, adolescents in high school may have to reduce the time for outdoor exercise and sleep and increase the time for near work, resulting in the heterogeneity of eye care behavior. Moreover, the results also suggested that there were significant differences in visual acuity development between the classes, with the prevalence of myopia and the number of people wearing glasses being higher in the bad and moderate behaviors class than in the healthy behaviors class. This finding is consistent with previous research. In 2014, the survey results showed that the ametropia of students in Beijing was significantly related to lower level activities (37). In 2021, Dutch researchers surveyed 525 teenagers' smartphone use, which also showed that refractive errors were significantly correlated with behaviors (38). Orlansky's findings suggest that poor vision affects a wide range of areas, such as reading, writing, posture, and movement, which may increase the likelihood of bad behavior in adolescents with poor vision (39). By identifying the characteristics of different latent behavior classes, students can be guided in a targeted way to protect their visual health.

## Conclusion

In summary, the results of the present study offer support for the notion that there is a diversity of eye care behaviors among adolescents. These subgroups also illustrate differential profiles in basic demographic characteristics and visual acuity development. In the future, a short and valid instrument may be developed accordingly to quickly screen and classify these subgroups. Eventually, we could expect an efficient and precise group intervention for students in different latent classes.

## Data Availability Statement

The raw data supporting the conclusions of this article will be made available by the authors, without undue reservation.

## Ethics Statement

This study was reviewed and approved by the Ethics Committee of the School of Public Health, Wuhan University. Written informed consent was obtained from the minor(s)' legal guardian/next of kin for the publication of any potentially identifiable images or data included in this article.

## Author Contributions

ML and BZ are responsible for manuscript writing and WW is responsible for data analysis. XT reviewed the manuscript. All the authors made substantial contributions to the completion of this manuscript, final approval of the version to be published, and agreed to be accountable for all aspects of the work.

## Conflict of Interest

The authors declare that the research was conducted in the absence of any commercial or financial relationships that could be construed as a potential conflict of interest.

## Publisher's Note

All claims expressed in this article are solely those of the authors and do not necessarily represent those of their affiliated organizations, or those of the publisher, the editors and the reviewers. Any product that may be evaluated in this article, or claim that may be made by its manufacturer, is not guaranteed or endorsed by the publisher.
